# Factors influencing value co-creation in cultural and creative enterprises: An empirical study

**DOI:** 10.1016/j.heliyon.2024.e35100

**Published:** 2024-07-23

**Authors:** Xiaodong Liu, Pei Liu, Meina Li

**Affiliations:** a Fashion. Art Design Institute, DongHua University, 1882 West Yan'an Road, Changning District, Shanghai, 200051, China; b Department of Mathematics and Physics, Faculty of Military Health Services, Naval Medical University, PLA Navy, 800 Xiangyin Road, Yangpu District, Shanghai, 200433, China; c Department of Military Medical Services, Faculty of Military Health Services, Naval Medical University, PLA Navy, 800 Xiangyin Road, Yangpu District, Shanghai, 200433, China

**Keywords:** Creative enterprise, Cultural enterprise, Customer value, Innovation capability, Value co-creation

## Abstract

The empirical validation of the relationship among the capabilities of cultural and creative enterprises (CCE), customer co-creation value, and enterprise value remains insufficient. Therefore, clarifying the essential capabilities for increasing enterprise and customer value is essential. This study explores the factors that influence value co-creation in cultural and creative enterprises and examines how this influences enterprise and customer value. To measure this, a structured questionnaire was distributed to cultural and creative practitioners in Shanghai, China, and AMOS 24.0 was used for structural equation modelling of the obtained survey data. The results confirm the positive impacts of cooperative innovation capability, customer-linking, and service capability on creating enterprise and customer value. Additionally, the results indicate an interactive relationship between enterprise value creation and customer co-creation value in this context. This study provides management insights for product innovation and to enhance customer service and competitive advantage.

## 1 Introduction

The advent of the creative-economy era has popularised research on cultural and creative industries [1,2]; however, studies show that the impact of cultural and creative industries on the global economy is underestimated [3], considering that economies will increasingly rely on creativity and innovation in the future [4]. Cultural and creative industries are organised sectors that focus on the creation, production and commercialisation of goods, services and activities with cultural, artistic or heritage characteristics [5]. Unlike other enterprises, the main assets of cultural and creative enterprises are intangible, such as creativity, know-how, information, services, and human and customer resources. Although a study on small- and medium-sized enterprises found that innovation had little impact on firm performance [6],the output of these enterprises—cultural and creative products—constitute a new type of independent market commodity. The characteristics of these products differ from those of traditional commodities. The focus of product design and operations management has shifted from production to creation. Additionally, cultural and creative enterprises (CCEs) often hold diverse portfolios of creative products and flexible, collaborative integration methods, which contribute to differences in efficiency and, ultimately, affect enterprise-value creation [7].

As an emerging business theory, the theory of value co-creation redefines the relationship between enterprises and customers, converting the traditional, one-way, value-transfer relationship into a process of value co-creation through interaction and cooperation [8]. With the current innovation and development, the roles of the enterprise and customer have changed, and the latter has become an active participant and creator of value realisation in addition to the role of consumer [9]. The theory of value co-creation emphasises that customers and enterprises participate in the entire process of project design, production, and consumption to jointly create value and enhance the experience. The process of value co-creation mainly includes interaction and co-operation between enterprises and customers, as well as co-creation among customers [8]. The theory of value co-creation can help enterprises to improve their service quality, reduce production costs, improve operational efficiency, and expand markets. From a customer's viewpoint, users can improve their satisfaction, enhance their recognition of the enterprise, and obtain a special experience by participating in the link of value co-creation. Based on the theory of value co-creation, this study explores the role generated in a CCE and related factors [10].

In CCE, customer value is based on Woodruff's widely quoted definition: 'a customer perceived preference for and evaluation of the products attributes, attribute performances, and consequences arising from use that facilitate (or block) achieving the customer's goals and purposes in use situations' [11]. Customer value includes expected value and value perception [12,13]. Expected value refers to customers' expectations for products or services. Customer value perception refers to the difference between customers' perceived total value from purchasing products or services and the total cost paid for them [14]. Perceived value is the value in the minds of customers. In most cases, customers are unaware of the actual production costs of the products they purchase; however, some customers intuitively feel that some products are more worthwhile based on their aesthetic value. Aesthetic value helps to tie customers in the context of cultural and creative products [15].

Furthermore, customers are more attentive to experiences while consuming cultural and creative products; thus, they participate in the design, production, and sales of these products [16]. In the relationship among CCE value creation, enterprise capability, and customer co-creation value, customer co-creation value is derived from reciprocal interaction between customers and suppliers to facilitate value [17]. In this context, capabilities play a vital role in creating value, as demonstrated by previous studies examining business-to-business relationships among enterprises [18]. Furthermore, creative capabilities are the primary source of an enterprise's competitive edge and longevity [19]. Research shows that customer linking and service align with service-dominant logic [20], promoting customer participation in value creation in service systems and thereby enhancing enterprise value creation and customer co-creation value [21,22]. Value creation in CCE should be based on customer maintenance, and customer co-creation value is regarded as the core purpose and key behaviour in market exchange [23]. In creating corporate value, CCE influences customers' value creation, which determines their experiences and perceptions [24]. Value co-creation is beneficial for customer relationships and to maintain the sustainable development of enterprises [25]. In addition, companies no longer create value independently but involve business partners and customers, thus realising value co-creation; however, little relevant research has been conducted [26]. Therefore, it is necessary to understand CCEs' capabilities to evaluate the contribution of value co-creation to enterprise-value creation and customer co-creation value.

Meanwhile, performance, as a core element of enterprise development, also affects an enterprise's degree of consumer awareness and willingness to buy. One study shows that business managers can effectively promote business performance by optimising customer satisfaction in practice, i.e. understanding customer needs and improving service quality can promote positive business development [27]. Customer engagement can also positively impact business performance. By optimising technology and developing relevant marketing strategies, companies can enhance customers' willingness to engage in and provide a driving force for business development [28]. Corporate sustainability, innovation performance, and environmental performance are also recognised as key factors in improving corporate performance, which can be effectively enhanced by optimising a company's organisational structure [29,30].

Recently, customers are viewed as consumers of products or services and as co-creators and co-producers [31]. Customers participate throughout value creation. Their co-creation experiences become the foundation of value. Gaining a competitive advantage in the future relies on the value co-creation process of customers and enterprises [32]. Although the interaction between customer co-creation value and enterprise value creation has attracted significant research, most studies focus on the retail, network, and service industries [33], while few studies examined CCE value. The dynamic relationship among CCE capabilities, customer co-creation value, and enterprise value creation has not been empirically validated. CCE and traditional enterprises differ regarding value creation and realisation methods, while intangible assets related to the knowledge and creativity of CCE constitute their unique capabilities. Currently, few previous studies address the relevant aspects of value co-creation in business development and explore its factors. Therefore, it is necessary to clarify the crucial capabilities in promoting the growth of enterprise-value creation and maintaining customer co-creation value for these enterprises. This requires identifying the measures that can be adopted to promote customers' active participation in the value-creation process. These are the prerequisites for CCEs to ensure sustained profitability. Therefore, elucidating such enterprises’ value-creation process requires establishing a framework model hypothesis and verifying the relationships among enterprise capability, enterprise-value creation, and customer co-creation value. The specific research questions of this study are as follows.

(1) How do firms utilise their capabilities to create co-creative value with their customers? Examining the above relationships provides useful insights, thus filling the gap in the literature on customer co-creativity.
(2) What are the causal relationships between firm capabilities, customer co-creation value, and firm-value creation?
(3) Which CCE's capabilities effectively contribute to customer co-creation value and corporate-value creation?

Based on the concept of value co-creation and related research, this study explores the impacts of antecedent and outcome variables while using interdisciplinary research methods to better understand consumer behaviour and value co-creation. The main contribution of this study lies in clarifying the impacts of technology and production, information technology, co-production, and other capabilities in enterprise development on customers' co-creation of value and enterprise-value creation, as well as the impact of value co-creation on an enterprise's sustainable development.

The rest of this study is structured as follows: Section 1 presents the literature review and research hypotheses; Section 2 presents the research methodology and data collection; Section 3 presents the data analysis and results; Section 4 presents the discussion and recommendations; and Section 5 presents the conclusion.

### 1.1 Relationship between CCE capability and enterprise value creation

Previous studies [34] demonstrated that an enterprise's capabilities enable it to conduct various activities; furthermore, multiple capabilities are pivotal in designing enterprises that integrate economic, environmental, and social sustainability harmoniously, thereby achieving a sustained competitive advantage that is difficult for competitors to replicate or surpass [35]. Enterprise capability is the skill that creates value for the enterprise and its customers. Almost every enterprise capability can produce tangible or intangible value. Due to the special characteristics of cultural and creative enterprises, their 'performance' is challenging to measure by traditional financial indicators [36]. Therefore, we assume that cultural and creative enterprises will pay attention to improving their innovation ability, service quality and other intangible assets in order to realise long-term value creation and customer value co-creation rather than overly pursuing short-term financial performance. CCE capabilities can be divided into technology, information, cooperation, product innovation, customer-linking and service, and co-integration [37].

The technological innovation capability of cultural and creative enterprises is achieved through production planning and scheduling, the expertise of technical teams, the development of patented technologies, and robust R&D capabilities. These elements combine to reduce marketing and service costs, minimise customer wait times, enhance service quality and interaction, and strengthen the security and privacy of consumer information. This enables them to be more competitive, customer-centric, and agile in the digital age, driving value creation and maintaining a market advantage. Studies reinforced the idea that technological innovation capabilities are critical drivers of value creation in cultural and creative enterprises, enabling them to stay competitive and customer-centric in a rapidly evolving digital landscape [[38], [39], [40]]. Another study also demonstrated that information technology capability improves enterprise performance, including financial and social performance [41,42]. Especially in emerging markets, high profitability and survival objectives of firms can be achieved through technological innovation [43]. Technological innovation in firms is recognised as a key factor in ensuring their competitiveness and improving their performance [44]. Technological innovation and business sustainability in enterprises are recognised as key factors in ensuring competitiveness and improving business performance [45,46]. In the turbulent world of business, technological innovation has become a major concern for top executives of companies [47].

The information technology capabilities of cultural and creative enterprises are instrumental in achieving a suite of strategic objectives that drive business success. These capabilities are honed through the application of IT to forecast trends in the cultural market, harness technology to predict potential opportunities, facilitate group and individual decision-making, and utilise IT to evaluate the continuation of partnerships. Previous studies explored the role of data mining in helping CCEs gain deeper insights into customer preferences, market trends, and operational data, enabling them to make informed decisions, optimise resource allocation, and improve the targeting of marketing and promotional activities, in turn, lead to enhancing the efficiency and effectiveness of resource integration within cultural and creative enterprises [48,49].

Cultural and creative enterprises can optimise their marketing and service processes through technological innovation, such as digital advertising, data analytics, and automation. This approach not only improves the efficiency of reaching target audiences but also leads to cost savings by streamlining marketing efforts. Additionally, implementing advanced customer relationship management systems and self-service options through technology enhances operational efficiency, thereby reducing service costs and minimising manual intervention.

Cooperative innovation capability in cultural and creative enterprises entails facilitating engagement with existing customers through technology platforms and attracting potential customers to participate in network-related activities [50]. Implementation of this capability requires developing user-friendly platforms, effective communication strategies, providing incentives for collaboration, and gathering feedback. Existing studies have suggested that enhancing cooperative innovation capabilities fosters collaborative relationships with consumers, yielding improved customer loyalty, enhanced brand reputation, and value co-creation [51]. Meanwhile, studies have shown that collaborative innovation capacity can promote product innovation and add impetus to the development of new products, which can enhance the core competitiveness of enterprises [51]. Previous studies have confirmed that customer-engagement behaviour positively impacts innovation, which is a core element of business development [52]. Some studies suggest a linear progression in the accumulation of cooperative innovation capability [53].

The customer-linking and service capabilities of CCE involve establishing specialised project teams, offering customised technical solutions based on customer needs, showcasing strong problem-solving skills, and upholding a stellar reputation among customers [54]. Empirical studies have investigated the correlation between these capabilities and outcomes, such as customer satisfaction, innovation, and the overall value proposition of CCE, providing solid evidence for the positive impact of customer-linking and service capabilities on value creation within the cultural and creative sector [[55], [56], [57]].

In addition, the integration capability of cultural and creative enterprises may play a key role in facilitating customer participation in value co-creation. The integration capability of enterprises can help them better integrate internal and external resources, optimise resource allocation, and improve operational efficiency [58,59]. Strong integration ability helps enterprises to establish close ties with customers, promote the interaction and information exchange between the two sides, and facilitate the creation of customer value [60,61].

Regarding the relationship between CCE capabilities and customer co-creation value, we propose the following hypotheses.

H1-1 The technological capability of CCE has a significant positive impact on customer co-creation value.

H1-2 The information technology capability of CCE has a significant positive impact on customer co-creation value.

H1-3 The cooperative production capacity of CCE has a significant positive impact on customer co-creation value.

H1-4 The cooperative innovation capability of CCE has a significantly positive impact on customer co-creation value.

H1-5 The customer-linking and service capability of CCE have a significant positive impact on customer co-creation value.

H1-6 The integration capability of CCE has a significant positive impact on customer co-creation value.

### 1.2 Relationship between CCE capability and customer Co-creation value

Customer co-creation value is an essential component of value creation, and it involves the joint efforts of businesses and customers to design innovative products, services, or experiences that satisfy their needs and preferences better. Customer co-creation value arises from the experience of consuming a product or service, primarily based on the aesthetic value of the interaction between customers and products [62]. An increase in customer co-creation value means that customers' expectations of the benefits have been met [63]. Therefore, enterprise capabilities are reflected through positive customer experience, naturally increasing customer co-creation value [64]. Enterprises can use their capabilities to create new customer co-creation value. Therefore, enterprise capabilities are linked to their capacity to deliver customer co-creation value. An enterprise's technological capability determines the degree of exquisiteness of its products [65]. The more exquisite the products, the higher the value perceived by customers. Additionally, customers' identification of a product's value depends on the information they obtain; thus, customer demand can be met by reducing information asymmetry between the product seller and the customer [66].

The harmonious integration of technological innovation capabilities within cultural and creative enterprises (CCEs) is instrumental in cultivating an environment that is ripe for customer co-creation. Empirical research has consistently underscored the critical role of technological innovation capabilities in enabling and enhancing customer co-creation. The benefits of this collaborative approach are multifaceted and include heightened customer satisfaction, a broader scope of product diversification, abbreviated wait times for customers, more competitive pricing of products, greater convenience in service delivery, and robust safeguards for data privacy and security [[67], [68], [69]]. Co-creation techniques are essential for business development; they can help companies provide more innovative services, and by proactively communicating with customers and engaging in social co-creation activities to gain access to innovative methods, necessary service improvements can be achieved [70]. Previous research has confirmed that firms influence consumers’ purchasing behaviour through value co-creation, and that firms can positively influence the co-creation of economic, enjoyment, and relational value through advanced technologies such as social-network markets [71]. These findings underscore the centrality of technological innovation as a catalyst for value creation and customer engagement within the cultural and creative industries.

Research indicates that strengthening the cooperative innovation capability of cultural and creative enterprises results in heightened levels of interaction between customers and enterprises [50]. This enhancement fosters increased customer engagement and participation in value co-creation activities, ultimately leading to the creation of tailored products, services, or experiences that elevate customer value [72]. Research confirms the positive role of the creative economy in generating employment and promoting firms’ innovation and creativity, which can contribute to value creation [73].

The customer-linking and service capabilities of cultural and creative enterprises are demonstrated through the establishment of specialised project teams to address unique customer needs, the provision of customised technical services, consistent problem-solving proficiency, and the maintenance of high levels of customer satisfaction with staff members. Empirical researches collectively reinforce the idea that the customer-linking and service capabilities of cultural and creative enterprises are instrumental in co-creating value with customers, ultimately contributing to business success and customer satisfaction that promotes customer value [67,[74], [75], [76], [77]]This is pertinent to our hypothesis that CCE's customer-linking and service capability significantly positively impact customer co-creation value.

In addition, enterprise management ability refers to the comprehensive ability of enterprises to formulate strategies, organise resources, lead decision-making, and promote implementation [78]. High-level management ability is the key factor for enterprises to continuously create value and maintain competitive advantage [79,80]. Excellent management ability helps enterprises formulate clear strategic objectives and development direction, as well as coordinate the effective implementation of various resources. The value creation of cultural and creative enterprises needs to organically combine innovation, marketing, service and other capabilities, which requires the management of enterprises to have systematic thinking and integration capabilities [81]. Artz et al. found that the management practice of enterprises has an important impact on their innovation performance [82]. High-level management ability can promote the efficient allocation of enterprise resources and provide necessary support for innovation activities. Enterprise management ability is also reflected in the optimisation of internal processes and the incentive and restraint of employees [83]. A reasonable organisational structure design, scientific decision-making process, and flexible incentive mechanism are all helpful in fully mobilising employees' work enthusiasm and improving work efficiency. Human resource management practices have a significant positive impact on performance indicators such as innovation and productivity of enterprises [84]. For knowledge-intensive cultural and creative enterprises, employees are the most valuable resources, and the scientific management of human resources by management is particularly critical [85]. At the same time, excellent management ability is also reflected in the coordination ability between enterprises and external stakeholders. The value creation of cultural and creative products often requires close cooperation between enterprises and suppliers, partners, customers and other stakeholders. Having a keen sense of market smell and good communication and coordination skills can help enterprises integrate external resources and create value together.

Therefore, we selected enterprise capability as a variable. Regarding the relationship between CCE capabilities and enterprise value creation, we propose the following hypotheses.

H2-1 The technological capability of CCE has a significant positive impact on enterprise value creation.

H2-2 The information technology capability of CCE has a significant positive impact on enterprise value creation.

H2-3 The cooperative production capability of CCE has a significant positive impact on enterprise value creation.

H2-4 The cooperative innovation capability of CCE has a significant positive impact on enterprise value creation.

H2-5 The customer-linking and service capabilities of CCE have a significant positive impact on enterprise value creation.

H2-6 The integration capability of CCE has a significant positive impact on enterprise value creation.

H2-7 The Management capacity of CCE has a significant positive impact on enterprise value creation.

### 1.3 Relationship between enterprise value and customer Co-creation value

Enterprise value creation is believed to increase with customer success [86]. Customer value growth can drive value growth for the enterprise [87]. Regarding the relationship between CCE value and customer co-creation value, we propose the following hypotheses.

H3 Customer co-creation value has a significant positive impact on enterprise value creation.

**Fig. 1:**
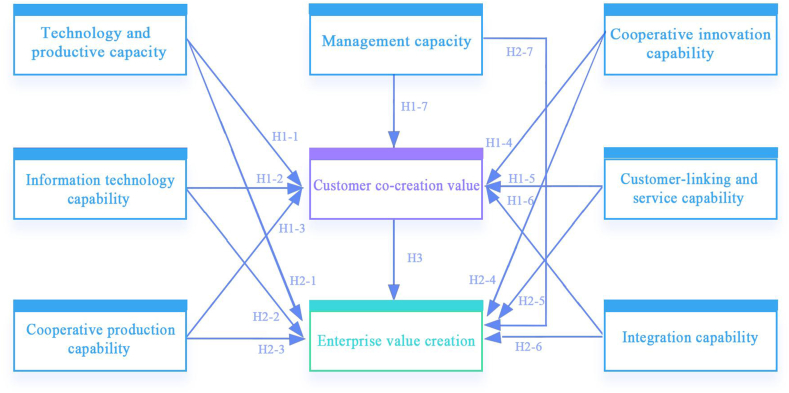
Generalised structural equation model.

Based on the above assumptions, the model constructed in this study is specified as follows in Fig. 1.

## 2 Materials and methods

### 2.1 Questionnaire and measure

A structured questionnaire was designed to verify the hypotheses. Questionnaire items were adapted from previous studies and developed through discussions with cultural and creative experts [88,89]. The questionnaire consisted of four parts. The first part focused on basic information on the surveyed enterprises, the second on CCE capabilities, the third on customer co-creation value, and the fourth on enterprise value creation (Supplement File S1).

Items related to customers' perceived value of cultural and creative products were taken from the customer perceived value scale, customer satisfaction scale, and customer co-creation values relative to the service context. Items of customer co-creation value increments for cultural and creative products were measured and analysed from the perspectives of economic, cultural, and social value. A 7-point Likert scale was used to measure the variables; responses ranged from 1 = 'strongly disagree' to 7 = 'strongly agree'.

### 2.2 Sampling and data collection

Questionnaires were sent to cultural and creative industry practitioners in Shanghai, China. The survey was performed from 3 to 20 April 2024. Nine parameters were estimated in the model, along with 15 arrowheads pointing at a latent variable. Following the prominent 10-times rule, the required sample size was determined to be at least 150 [90]. A total of 371 survey questionnaires were distributed, and 363 were returned. Of the returned questionnaires, 31 were excluded owing to incomplete information. Thus, 332 valid questionnaires were analysed, with an effective response rate of 91.46 %.

### 2.3 Data entry and statistical analysis

Two authors entered the data using Epidata 3.1 software and analysed them using SPSS software. We used descriptive statistics to describe the respondents' basic information. The *t*-test was used to test the sample's stability. We employed exploratory factor analysis (EFA) to test the reliability and validity of the questionnaire. The internal consistency of the variables was measured by Cronbach's alpha with a value of 0.7, indicating high internal consistency [91]. Linear Structural Relations 8.73, a specialised structural equation module, was used to test the construct validity of the questionnaire through confirmatory factor analysis. Using factors with an eigenvalue ≥1, an orthogonal rotation was performed via the maximum variance method. The item factor loading of >0.5 was chosen to ensure the questionnaire had good structural validity.

Based on survey reliability and validity tests, AMOS 24.0 was used for structural equation modelling. We used a structural equation model to capture the measured variables and define the degree of latent variables and their relationship.

### 2.4 Ethics statement

The study was conducted and approved by the Science and Technology Ethics Committee, Donghua University (DHUEC-FZ-2024-02). Informed consent was obtained from all subjects involved in the study.

## 3 Results

### 3.1 Basic characteristics

Table 1 shows that most respondents were under 30 years of age (59.14 %). Most respondents had completed high school (92.77 %), while 57.53 % had a bachelor's degree and 35.24 % had a master's degree or above. Regarding the enterprise category, culture and art accounted for 21.69 %, and design services accounted for 11.44 %. Most enterprises were older than 15 years (51.81 %), and 31.93 % had more than 200 employees. The largest number of respondents worked in their enterprise's creative department (26.81 %), followed by those working in the Financial department (10.84 %) and research and development (R&D) design (10.54 %).

**Table 1:** Demographic characteristics.

Characteristics	Frequency	%
**N**	332	100.0
**Age**		
≤20	60	18.07
21–30	136	40.97
31–40	53	15.96
41–50	49	14.76
51–60	32	9.64
≥61	2	0.60
**Education**		
High school	24	7.23
Bachelor's degree	191	57.53
Master's degree and above	117	35.24
**Department**		
Manufacturing	14	4.22
Information department	50	15.06
Creative department	89	26.81
Human resources department	9	2.71
Quality control department	17	5.12
Financial department	36	10.84
Marketing/business department	15	4.52
R & D design	35	10.54
Purchasing department	7	2.11
Administration	2	0.60
Other	58	17.47
**Position**		
General manager	9	2.71
Deputy general manager	18	5.42
Department manager	57	17.17
Employee	106	31.93
Other	142	42.77
**Category of company**		
Culture and art	72	21.69
News publishing	23	6.93
Radio, TV, and movies	21	6.32
Software, network, and computer services	17	5.12
Advertising exhibition	22	6.63
Art trade	25	7.53
Design service	38	11.44
Travel and recreation	42	12.65
Other services	72	21.69
**Established time**		
5 years or less	44	13.25
5–10 years	72	21.69
10–15 years	44	13.25
15 years or more	172	51.81
**Company size**		
50 people or less	84	25.30
50–100 people	86	25.90
101–200 people	56	16.87
More than 200 people	106	31.93

### 3.2 Evaluation of measurement models

In this study, the reliability and validity of the collected questionnaires were analysed using SPSS software. According to Table 2, it can be concluded that the KMO test is 0.919 (the permissible interval is between 0 and 1, and the closer it is to 1, the better the validity of the questionnaires of the present study). According to Bartlett's test, the P-value is infinitely close to 0, indicating that this study is reliable.

**Table 2:** KMO and Bartlett's inspection.

Fit indices	Chi-square/df	RMSEA	NFI	IFI	CFI
Actual	1.177	0.034	0.927	0.988	0.988
Recommended	＜3	＜0.06	＞0.85	＞0.85	＞0.85

In this study, CFA (Confirmatory Factor Analysis) was applied to test the model, and the results are shown in Table 3; the overall fit of the model is good, and all the data meet the specification requirements. Chi-squard/df = 1.177 (recommended value with between 1.0 and 3.0); RMSEA is 0.034, which is lower than the specification requirement of 0.06; NFI = 0.927, IFI = 0.988, and CFI = 0.988 were all higher than the normative requirement of 0.85. Based on the results of the data analysis in Tables 3 and it is clear that all the data are within acceptable limits, and therefore the model has a good fit.

**Table 3:** The values of fit indices.

Fit indices	Chi-square/df	RMSEA	NFI	IFI	CFI
Actual	1.177	0.034	0.927	0.988	0.988
Recommended	＜3	＜0.06	＞0.85	＞0.85	＞0.85

In order to assess the reliability and validity of the data, Cronbach's alpha, analysis of reliability (CR) and average variance of extraction (AVE) were calculated in this study. The results are shown in Table 4; all the alpha coefficients in this study were in the range of 0.847–0.943, which meets the requirement of being greater than 0.7 [91]The values of CR in this study were all above 0.7, indicating good agreement between the variables. The values of AVE in this study ranged from 0.685 to 0.806, indicating that our study is valid.

**Table 4:** Results of confirmatory factor analysis.

Items and contents	Factor loading	AVE	CR	α
1. Technology and productive capacity				
1.1. Market share of cultural and creative enterprises	0.789	0.685	0.897	0.897
1.2. Sales channels of cultural and creative enterprises	0.82			
1.3. Workflow of cultural and creative enterprises	0.832			
1.4. Customer development and product services	0.868			
2. Information technology (IT) capability				
2.1. Enterprises use information technology (IT) to predict trends in the cultural market	0.947	0.788	0.937	0.936
2.2. Enterprises use IT to identify potential opportunities in the cultural market in advance	0.873			
2.3. Enterprises use IT to collect information on cultural market research	0.861			
2.4. Enterprises use IT to support transactions between enterprises and customers	0.867			
3. Cooperative production capability				
3.1. The degree of information exchange between enterprises and their partners	0.889	0.759	0.927	0.926
3.2. The degree of mutual capital dependence when the enterprises hold shares in their cooperative manufacturers	0.852			
3.3. The degree of mutual capital dependence between the enterprises and their cooperative manufacturers when circulating funds	0.868			
3.4. The degree of mutual interaction between enterprises and their cooperative enterprises when dispatching personnel to visit each other	0.876			
4. Cooperative innovation capability				
4.1. Improving the rapid response of cultural and creative services	0.932	0.806	0.943	0.943
4.2. Being able to improve products based on consumer purchase data	0.906			
4.3. Strengthening the channel advantages of cultural and creative products	0.867			
4.4. Increasing the profitability of cultural and creative brand extensions	0.885			
5. Customer-linking and service capability				
5.1. Services of enterprises aimed at improving customer satisfaction	0.818	0.638	0.876	0.875
5.2. Providing consumers with diversified product choices	0.807			
5.3. Reducing customer waiting time	0.752			
5.4. Reducing the price of cultural and creative products	0.817			
6. Integration capability				
6.1. Enterprises simulate customer demand levels to ensure stable operations and the utilization of production capacity	0.794	0.583	0.848	0.847
6.2. Enterprises can still complete double orders on time to meet customer needs	0.729			
6.3. Enterprises can share intangible costs, such as advertising and R&D through substantially large sales	0.769			
6.4. Enterprises will mass-produce based on their advantages to reduce costs	0.761			
7.Management capacity				
7.1 The management capabilities of the business, organisational structure, decision-making processes, leadership and the management and motivation of employees.	0.777	0.672	0.891	0.89
7.2 The efficiency of a company's daily operational activities such as productivity, supply chain management, logistics and distribution, and customer service.	0.839			
7.3 The level of expertise of the enterprise in a given technological field, including the ability to develop, produce and apply the technology.	0.836			
7.4The soft power of the company's values, mission, working atmosphere and social responsibility.	0.825			
8. Customer co-creation value				
8.1. Enterprises can link different independent customers to form a value network	0.827	0.682	0.914	0.909
8.2. Enterprise can integrate with other enterprises to form a value creation network	0.81			
8.3. Enterprises can communicate within the cultural industry and with different industrial networks	0.789			
8.4. Enterprises' technology platform is available to all customers related to enterprises	0.8			
8.5 Enterprises can offer customised services to users	0.898			
9. Enterprise value creation				
9.1. Enterprises gather talents from different professional fields to solve customer problems	0.812	0.702	0.904	0.909
9.2. Enterprises will quickly meet customer expectations based on specific goals that customers are interested in	0.845			
9.3. Enterprises will set up a project team to address customer special cases	0.826			
9.4. Enterprises will provide services of different technical levels according to the different levels of customer problems	0.867			

### 3.3 Hypotheses testing

**Fig. 2:**
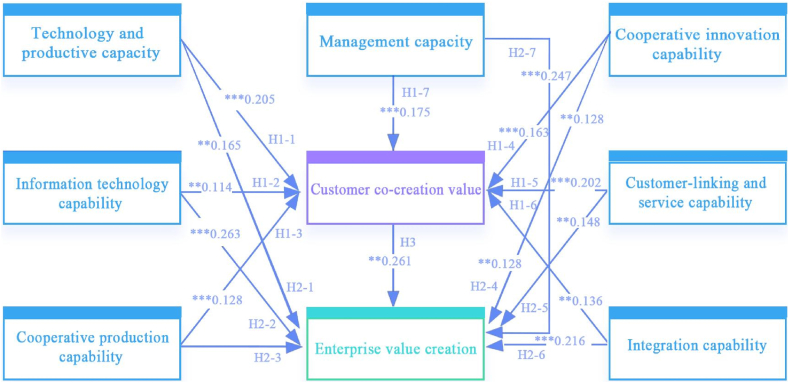
Path analysis results.

Table 5 and Fig. 2 sort out the research paths of this study, and P in the table indicates the significance of the paths (*** when P < 0.001, "**" when P is between 0.001 and 0.01, "*" when P is between 0.01 and 0.05, "* ", otherwise not significant) [92]. The results show that Technology capability has a significant effect on Customer co-creation value (H1-1: β = 0.25, p < 0.001). Information technology capability has a significant effect on Customer co-creation value (H1-2: β = 0.153, P = 0.003) has a significant effect. Cooperative production capability has a significant effect on Customer co-creation value (H1-3: β = 0.186, P < 0.001). Cooperative innovation capability has a significant effect on Customer co-creation value (H1-4: β = 0.249, p < 0.001). Customer linking and service capability have a significant effect on Customer co-creation value (H1-5: β = 0.249, p < 0.001). Value (H1-5: β = 0.259, P=<0.001) has a significant effect. Integration capability has a significant effect on Customer co-creation value (H1-6: β = 0.158, P = 0.005). Management capability has a significant effect on Customer co-creation value (H1-7: β = 0.207, P < 0.001) has a significant effect. Technology capability has a significant effect on Enterprise value creation (H2-1: β = 0.159, P = 0.003). Information technology capability has a significant effect on Enterprise value creation (H2-2: β = 0.282, P < 0.001). The hypothesis of cooperative production capability has a significant effect on Enterprise value creation (H2-3: β = −0.012, P = 0.82) was not supported. Cooperative innovation capability has a significant effect on Enterprise value creation (H2-4: β = 0.282, P < 0.001). Customer linking and service capability have a significant effect on Enterprise value creation (H2-5: β = 0.151, P = 0.006). Integration capability has a significant effect on Enterprise value creation (H2-6: β = 0.199, P < 0.001) has a significant effect. Management capability has a significant effect on Enterprise value creation (H2-7: β = 0.233, P < 0.001). Customer co-creation value has a significant effect on Enterprise value creation (H3: β = 0.207, P = 0.002) has a significant effect.

**Table 5:** Results of hypotheses testing.

Hypothesis	Estimate	S.E.	C.R.	P	β	Hypotheses Verification
H1-1 Technology capability→ Customer co-creation value	0.205	0.046	4.508	***	0.25	Supported
H1-2 Information technology capability→ Customer co-creation value	0.114	0.039	2.923	0.003	0.153	Supported
H1-3 Cooperative production capability→ Customer co-creation value	0.128	0.037	3.481	***	0.186	Supported
H1-4 Cooperative innovation capability→ Customer co-creation value	0.163	0.035	4.68	***	0.249	Supported
H1-5 Customer-linking and service capability→ Customer co-creation value	0.202	0.044	4.61	***	0.259	Supported
H1-6 Integration capability→ Customer co-creation value	0.136	0.048	2.832	0.005	0.158	Supported
H1-7 Management capability→ Customer co-creation value	0.175	0.046	3.763	***	0.207	Supported
H2-1 Technology capability→ Enterprise value creation	0.165	0.056	2.933	0.003	0.159	Supported
H2-2 Information technology capability→ Enterprise value creation	0.263	0.048	5.485	***	0.282	Supported
H2-3 Cooperative production capability→ Enterprise value creation	−0.01	0.045	−0.228	0.82	−0.012	NOT Supported
H2-4 Cooperative innovation capability→ Enterprise value creation	0.128	0.043	2.97	0.003	0.156	Supported
H2-5 Customer-linking and service capability→ Enterprise value creation	0.148	0.054	2.736	0.006	0.151	Supported
H2-6 Integration capability→ Enterprise value creation	0.216	0.059	3.658	***	0.199	Supported
H2-7 Management capability→Enterprise value creation	0.247	0.058	4.284	***	0.233	Supported
H3 Customer co-creation value→ Enterprise value creation	0.261	0.083	3.143	0.002	0.207	Supported

## 4 Discussion

The research findings support the idea that cooperative innovation capability, customer-linking, and service capabilities of CCE enhance enterprise performance and positively impact enterprise value. Firstly, cooperative innovation capability enables cultural and creative enterprises to engage customers in the innovation process, co-creating value through collaborative product development and customisation. Secondly, customer-linking capability allows enterprises to establish strong relationships with customers, facilitating the exchange of knowledge and resources that are essential for value co-creation. Finally, service capability empowers enterprises to deliver high-quality, personalised experiences that enhance customer satisfaction and loyalty, leading to increased customer participation in value co-creation. These findings are similar to those of Lee, who revealed that enterprise capability is the primary source of a company's competitive edge [93], and those of O'Cass and Ngo, who found that cooperative innovation capability is a significant factor in competitiveness [94]. These capabilities enable companies to adapt their strategies more flexibly in a changing market and technological environment, quickly adapting to changes in the business and meeting the needs of sustainable development in the market. Previous studies demonstrated a positive relationship between customer-linking service capability and customer relationship performance [95], which can improve customer satisfaction. For CCE, customer-linking and service capabilities are critical success factors for achieving customer loyalty and enterprise development [96]. Accordingly, in the mature phase of their life cycle, CCE pays more attention to cooperative innovation and dedicates more resources to innovation for better development [92].

Previous research explored the technological and information capabilities of industries. Afuah estimated an enterprise's customer value and competitive advantage based on its technological capabilities in the market for cholesterol drugs [97]. Navimipour et al. showed that information technology plays a significant role in enhancing the performance of an enterprise [98]. Our research further validates this idea that technology and production capabilities and IT capabilities can, to a certain extent, contribute to enterprise value creation and customer co-creation of value. As companies enhance their technology and adopt advanced technologies such as artificial intelligence, cloud computing, and big data processing, they can optimise their operational processes and improve overall operational efficiency [99]. At the same time, as the depth of research and development increases, technology enables companies to develop new products more quickly and develop the latest solutions to meet the needs of the market; companies continue to innovate [99].

Previous studies also found that an enterprise's integration capabilities do not moderate efficiency or effectiveness [100]. Then, the findings of this study are the opposite, which may be due to the fact that with the optimisation of the integration capability of enterprises, they are able to make more effective use of related resources such as capital, talent and technology, and strive to improve the efficiency of resource allocation, thus enhancing the operational performance of enterprises. At the same time, enterprises with strong integration capabilities can also better identify and take advantage of market opportunities and, through the optimisation of resource allocation, are able to form their competitive advantages, enhance their market position, and promote enterprise transformation and upgrading.

The findings of this study show that cooperative production capacity has little effect on enterprise value creation. This is contrary to the findings of previous studies. This may be due to the fact that cooperative production may have some kind of uncertainty that increases the operational risk assumed by the enterprise. Especially in the face of the current situation of high market volatility, entering into cooperative relationships with enterprises that do not understand it may have a negative impact on the development of the firm.

This study also found that the management capability of an enterprise also contributes to enterprise value creation and customer co-creation capability to some extent. An excellent enterprise management strategy can ensure that the enterprise develops a clear, long-term strategic plan, which is conducive to clarifying the enterprise's future development put down, and optimising the allocation of resources so as to coordinate the enterprise's value creation ability [86]. Strong enterprise management ability can promote enterprise innovation in product development, service, and process; through the development of reasonable management strategy, enterprises can improve operational efficiency, enhance the core competitiveness of enterprises and enhance market competitiveness. At the same time, enterprise management can also coordinate the relationship with customers; enterprises can better explore customer needs and provide flexible and controllable personalised services, which in turn pay attention to improve customer satisfaction and loyalty and promote customer co-creation of value [86].

In their subsequent operations, enterprises should pay more attention to the importance of technological and production capacity and information technology capabilities, which were found to have the greatest impact on value creation and customer co-creation. Enterprises should continuously enhance their internal innovation capability, strengthen their R&D depth, utilise technology and information acquisition capability to continuously enhance value creation within the enterprise, and promote interaction and co-creation with customers. At the same time, the ability to cooperate and innovate is also important. Moreover, in terms of practical impact, despite the promises, it is not clear how the findings can help firms that lack resources, support, and capabilities. Still, this study provides insights for cultural and creative firms that lack resources and capabilities. Firstly, they should prioritise the development of collaborative innovation capabilities, customer linkages, and service capabilities, as these have the greatest impact on firm value creation and customer value co-creation. Secondly, they can cooperate with partners who have advantages in technology and information technology to make up for their shortcomings with external resources. Finally, through continuous capacity building, they can gradually consolidate their basic capabilities in technology and information technology so as to comprehensively enhance their capabilities in value creation and value co-creation.

## 5 Conclusions

Our findings enhance the current understanding of how enterprise capability ensures value co-creation in CCE. Cooperative innovation capability, customer-linking, and service capability of CCE were found to have strong and positive effects on enterprise value and customer co-creation value. These values also have positive effects on each other. The findings of this study suggest that facilitating joint value creation between enterprises and customers is essential to ensure a successful collaborative relationship. Furthermore, CCE can improve its competitive advantage through targeted capacity-building activities to improve its cooperative innovation, customer-linking, and service capabilities.

## Limitations and recommendations for future research

Value co-creation is an important aspect of enterprise development; this study explores the relationship between CCEs' capabilities, customers' co-creation value, and enterprise value. Moreover, it provides relevant insights for enterprises' product innovation, improving their service quality, and enhancing their competitive advantage. However, some limitations in this study must be considered in future research. First, this study only explores the impacts of firms' different capabilities on value co-creation, and more variables can be explored in future studies for their impacts on value co-creation, such as green innovation, firm performance, and others. Second, the subjects investigated in this study are mostly Chinese firms; future studies can extend the study group to international firms while conducting quantitative comparative studies. For example, they can explore whether there are differences in the value co-creation models practiced by enterprises in different countries. Finally, with the current development, enterprises' operation mechanism will also be constantly innovated, and it is suggested that subsequent research explore the development trend of value co-creation over a longer time horizon and examine how to balance the relationship between customers and enterprises so as to promote enterprises’ sustainable development.

## Funding

This work was supported by the 10.13039/501100001809National Natural Science Foundation of China (grant number 72274209), 2024 Shanghai Municipal Project for Promoting the Development of Cultural and Creative Industries (No. 2024020004-V0), the Shanghai Art Science Planning Project (grant number YB2022–F-059), the Fundamental Research Funds for the Central Universities (grant number 2232021B-03), 2023 Arts and Crafts Research Project of China Arts and Crafts Society (grant number CNACS2023-I-38), and the Soft Science Project of Shanghai Science and Technology Innovation Action Plan (grant number 23692113200).

## Data availability statement

The data used to derive the findings of this study are available from the corresponding author upon request.

## CRediT authorship contribution statement

**Xiaodong Liu:** Writing – review & editing, Supervision, Resources, Project administration, Investigation, Data curation, Conceptualization. **Pei Liu:** Writing – original draft, Visualization, Software, Methodology, Formal analysis. **Meina Li:** Writing – original draft, Validation, Funding acquisition, Conceptualization.

## Declaration of competing interest

The authors declare that they have no known competing financial interests or personal relationships that could have appeared to influence the work reported in this paper.
